# Chlorogenic Acid: Recent Advances on Its Dual Role as a Food Additive and a Nutraceutical against Metabolic Syndrome

**DOI:** 10.3390/molecules22030358

**Published:** 2017-02-26

**Authors:** Jesús Santana-Gálvez, Luis Cisneros-Zevallos, Daniel A. Jacobo-Velázquez

**Affiliations:** 1 Tecnológico de Monterrey, Escuela de Ingeniería y Ciencias, Centro de Biotecnología FEMSA, Av. Eugenio Garza Sada 2501 Sur, C.P. 64849 Monterrey, Mexico; jsantanag2000@gmail.com; 2 Department of Horticultural Sciences, Texas A&M University, College Station, TX 77843-2133, USA; lcisnero@tamu.edu

**Keywords:** chlorogenic acid, 5-*O*-caffeoylquinic acid, metabolic syndrome, antibiotic, antioxidant, prebiotic

## Abstract

Chlorogenic acid (5-*O*-caffeoylquinic acid) is a phenolic compound from the hydroxycinnamic acid family. This polyphenol possesses many health-promoting properties, most of them related to the treatment of metabolic syndrome, including anti-oxidant, anti-inflammatory, antilipidemic, antidiabetic, and antihypertensive activities. The first part of this review will discuss the role of chlorogenic acid as a nutraceutical for the prevention and treatment of metabolic syndrome and associated disorders, including in vivo studies, clinical trials, and mechanisms of action. The second part of the review will be dealing with the role of chlorogenic acid as a food additive. Chlorogenic acid has shown antimicrobial activity against a wide range of organisms, including bacteria, yeasts, molds, viruses, and amoebas. These antimicrobial properties can be useful for the food industry in its constant search for new and natural molecules for the preservation of food products. In addition, chlorogenic acid has antioxidant activity, particularly against lipid oxidation; protective properties against degradation of other bioactive compounds present in food, and prebiotic activity. The combination of these properties makes chlorogenic acid an excellent candidate for the formulation of dietary supplements and functional foods.

## 1. Introduction

**Figure 1:**
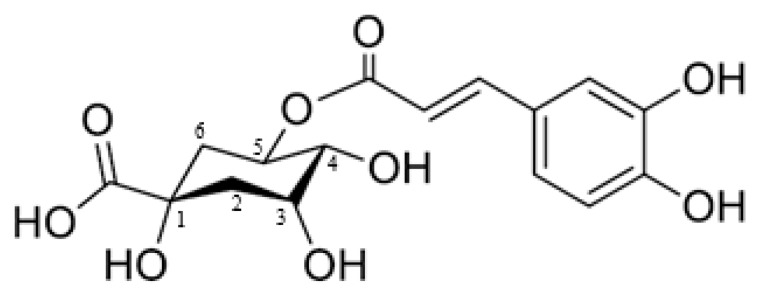
Chemical structure of chlorogenic acid (5-*O*-caffeoylquinic acid).

Chlorogenic acid is a phenolic compound from the hydroxycinnamic acid family. The compound’s chemical structure consists of a caffeic acid moiety and a quinic acid moiety; therefore, it is also known as 5-*O*-caffeoylquinic acid (5-CQA), although some authors also refer to it as 3-CQA [1,2], a nomenclature discrepancy closely linked to the history of chlorogenic acid [3]. However, according to the International Union of Pure and Applied Chemistry (IUPAC) current numbering rules [4], it should be named 5-CQA. The chemical structure and current IUPAC numbering of chlorogenic acid are shown in Figure 1.

Chlorogenic acid has been extensively studied since it is widely distributed in plants, is one of the main polyphenols in the human diet, and it possesses many health-promoting properties. Chlorogenic acid can be found in foods and herbs such as apples [5,6,7,8,9], artichoke [10], betel [11], burdock [12], carrots [13,14,15], coffee beans [5,7,8,9,11], eggplants [5], eucommia [16], grapes [8], honeysuckle [7], kiwi fruit [9], pears [5], plums [5,6], potatoes [5,7,17,18], tea [8,11], tobacco leaves [5], tomatoes [5], and wormwood [19]. Furthermore, it has been found that chlorogenic acid has anti-oxidant [20], anti-inflammatory [20], anticancer [21,22,23,24,25], antilipidemic [26], antidiabetic [26], antihypertensive [27], and antineurodegenerative activities [28,29,30]. Moreover, most research regarding the health benefits of chlorogenic acid has been done on disorders related to metabolic syndrome, which is defined as a group of interconnected physiological, biochemical, clinical, and metabolic factors that increase the risk of cardiovascular diseases, type 2 diabetes mellitus, and all-cause mortality [31]. It has been estimated that 25% of the world’s adult population has this syndrome [32]. In addition, the syndrome is considered a worldwide epidemic with high socioeconomic cost and increasing prevalence in both childhood and young adulthood [33]. Therefore, the first part of this review will discuss the role of chlorogenic acid as a nutraceutical for the prevention and treatment of metabolic syndrome and associated disorders, including in vivo studies, clinical trials, and mechanisms of action.

The second part of the review will be dealing with the role of chlorogenic acid as a food additive. Chlorogenic acid has activity against a wide range of microorganisms, including bacteria, yeasts, molds, viruses, and amoebas. These antimicrobial properties can be useful for the food industry in its constant search for new and natural molecules for the preservation of food products. Also, the antioxidant properties of chlorogenic acid have shown promise for the conservation of foods, particularly for inhibition of lipid oxidation. Finally, this phenolic has demonstrated protective properties against degradation of other bioactive compounds, as well as prebiotic activity.

## 2. The Role of Chlorogenic Acid as a Nutraceutical for the Prevention and Treatment of Metabolic Syndrome and Associated Disorders

Metabolic syndrome is a complex, multifactor disorder that increases the risk of cardiovascular diseases and diabetes mellitus type 2. Its components include dyslipidemia (elevated plasma total cholesterol, low-density lipoproteins (LDL), and triglycerides, and low levels of high-density lipoproteins (HDL)), high blood pressure, high fasting blood glucose levels, insulin resistance, chronic proinflammatory and prothrombotic states, non-alcoholic fatty liver, and sleep apnea [33]. Obesity has been acknowledged as the main cause of this syndrome [34]. Currently, there is no single treatment for metabolic syndrome, and interest in natural products as potential treatments has increased [35]. Many studies have been done to evaluate the effect of chlorogenic acid on health, where most of them involve metabolic syndrome or associated disorders, including obesity, dyslipidemia, diabetes, and hypertension. In vivo studies and clinical trials are summarized in Table 1 and Table 2, respectively.

### 2.1. Obesity

#### 2.1.1. In Vivo Studies

Studies in animals have demonstrated that chlorogenic acid has activity against obesity. Cho et al. [36] investigated the effect of chlorogenic acid on body weight, body fat, and obesity-related hormones of induced-obese imprinting control region (ICR) mice. The 32 male mice were divided into four groups and were fed a normal diet, a high-fat diet (37% calories from fat), a high-fat diet with 0.02% (0.2 g/kg diet) of chlorogenic acid, or a high-fat diet with 0.02% caffeic acid for 8 weeks. Both chlorogenic acid and caffeic acid significantly lowered (*p* < 0.05) body weight, visceral fat mass, plasma leptin and insulin levels, triglycerides in liver and heart, and cholesterol in adipose tissue and heart compared to the high-fat control group. In addition, only chlorogenic acid significantly lowered triglyceride contents in adipose tissue. Overall, chlorogenic acid seemed to be more effective than caffeic acid. Huang et al. [37] obtained similar results in rats. Forty male Sprague-Dawley rats were divided into four groups and were fed a normal diet, a high-fat diet, a high-fat diet with a low dose of chlorogenic acid (20 mg/kg body weight (BW)), or a high-fat diet with a high dose of chlorogenic acid (90 mg/kg BW). Chlorogenic acid was dissolved in sterile saline and administered orally by gavage once daily for 12 weeks, while the normal and high-fat control groups were given sterile saline. Chlorogenic acid suppressed in dose-dependent manner increases in body and visceral fat weights, and hepatic free fatty acids induced by high-fat diet.

#### 2.1.2. Clinical Trials

Human studies have also revealed anti-obesity effects of chlorogenic acid-rich foods. Thom [45] gave to 30 overweight subjects for 12 weeks either five cups of normal instant coffee per day (equivalent to 11 g of coffee per day), or five cups of Coffee Slender^®^ per day, the latter being rich in chlorogenic acid and its isomers (~45 mg/g, 33% of each isomer). Participants showed a significant reduction in weight (*p* < 0.05; −5.4 kg) with Coffee Slender^®^, where 80% of the reduction was due to loss of body fat. On the other hand, the participants who drank normal instant coffee showed non-significant reductions in body weight and body fat. Moreover, Soga et al. [46] investigated how chlorogenic acid and other related compounds such as isomers, derivatives, and/or other quinic acid conjugates (further referred as CGAs) influenced energy metabolism. Eighteen healthy male subjects (36.1 ± 7.4 years old) consumed 185 mL of a test beverage with or without CGAs (329 mg) daily for four weeks. Indirect calorimetry showed that the CGAs beverage led to a significantly higher (*p* < 0.05) postprandial energy expenditure compared to the control beverage. Also, the subjects who ingested the CGAs beverage exhibited higher postprandial fat utilization.

### 2.2. Dyslipidemia

The effect of chlorogenic acid on dyslipidemia has only been evaluated in animals. The obesity in vivo studies earlier described (see Section 2.1.1) found positive effects of chlorogenic acid over dyslipidemia. Cho et al. [36] observed that both chlorogenic acid and caffeic acid significantly lowered (*p* < 0.05) in mice plasma free fatty acids, triglycerides, and cholesterol, and significantly increased HDL-cholesterol/total cholesterol ratio compared to the high-fat control group. Moreover, Huang et al. [37] found that chlorogenic acid suppressed in dose-dependent manner the serum lipid levels induced by high-fat diet. In addition, Wan et al. [38] studied the effect of chlorogenic acid on hypercholesterolemia in rats. Forty male Sprague-Dawley rats were divided in four groups and were given a normal diet, a high-cholesterol diet, a high-cholesterol diet with a low dose of chlorogenic acid (1 mg/kg BW/day), or a high-cholesterol diet with a high dose of chlorogenic acid (10 mg/kg BW/day). Chlorogenic acid was administered orally by gavage (20 mL/kg BW) once per day for 28 days, while control groups were given distilled water. It was found that chlorogenic acid administered in a high dose (10 mg/kg BW) significantly reduced total and LDL-cholesterol (*p* < 0.05), and increased HDL cholesterol (*p* < 0.01). Furthermore, chlorogenic acid improved both the atherogenic index and the cardiac risk factor. Studies in humans are still needed to evaluate the effect of chlorogenic acid over dyslipidemia, i.e., levels of serum total cholesterol, LDL, and HDL.

### 2.3. Diabetes

#### 2.3.1. In Vivo Studies

Chlorogenic acid has shown beneficial effects against diabetes in animals regarding blood glucose levels, lipid metabolism, cataracts, and wound- healing. Ong et al. [39] randomly assigned 20 *Lepr^db/db^* mice into five groups and four C57BL/6 mice were assigned as control group. The mice were treated daily for 2 weeks with vehicle, 250 mg/kg BW metformin by oral gavage or 250 mg/kg BW chlorogenic acid intraperitoneally. Chlorogenic acid inhibited hepatic glucose-6-phosphatase expression and activity, decreased hepatic steatosis, improved lipid profiles and skeletal muscle glucose uptake, which improved fasting glucose levels, glucose tolerance, insulin sensitivity, and dyslipidemia in the *Lepr^db/db^* mice. Furthermore, Hunyadi et al. [40] investigated the antidiabetic activity of an extract of the leaves of the white mulberry tree (*Morus alba* L.) and three of its major constituents (chlorogenic acid, rutin, and isoquercetin) in rats. Newborn Sprague-Dawley rats were treated with 150 mg/kg BW streptozotocin intraperitoneally to induce diabetes. Eight weeks later, the rats were divided in groups of six or seven animals and were orally treated once a day for 11 days with mulberry extract (250 or 750 mg/kg BW), chlorogenic acid (9 or 27 mg/kg BW), rutin (5 or 15 mg/kg BW), or isoquercetin (3 or 9 mg/kg BW) suspended in 0.25% of methylcellulose using a dosing volume of 5 mL/kg BW for all treatments. A dose-dependent decrease of non-fasting blood glucose levels was found for mulberry extract, chlorogenic acid (27 mg/kg BW) and rutin, but not for isoquercetin. Chlorogenic acid and rutin were accounted for as much as half the observed antidiabetic activity of mulberry extract.

Jin et al. [16] examined the effect of chlorogenic acid on glucose and lipid metabolism in late diabetic mice. Thirty-two female C57BL/BKS mice were divided in four groups: *db*/*m*-control group, *db*/*m*-chlorogenic acid group, *db*/*db*-control group, and *db*/*db*-chlorogenic acid group. Animals in the chlorogenic acid groups were treated once daily with chlorogenic acid (80 mg/kg BW) for 12 weeks by lavage, while controls were given phosphate-buffered saline (PBS). Compared to the *db*/*db*-control group, the percentage of body fat, fasting plasma glucose, and glycosylated hemoglobin (HbA1c) significantly decreased (*p* < 0.05) in the *db*/*db*-chlorogenic acid group. In addition, Kim et al. [41] used a galactose-fed rat model to determine the influence of chlorogenic acid on sugar cataracts. Forty male Sprague-Dawley rats were randomized into five groups, and were treated with a normal diet, 50% galactose diet, 50% galactose diet with low dose of chlorogenic acid (10 mg/kg BW), or 50% galactose diet with high dose of chlorogenic acid (50 mg/kg BW). Chlorogenic acid was administered orally once a day for two weeks. It was found that chlorogenic acid prevented the development of sugar cataracts.

Finally, Bagdas et al. [42] studied the impact of chlorogenic acid treatment on diabetic wound healing in rats. Forty male Sprague-Dawley rats were intraperitoneally injected with 45 mg/kg BW streptozotocin to induce diabetes, and wounds were created on their backs. The animals were divided in two groups, where 50 mg/kg BW of chlorogenic acid or PBS were administered intraperitoneally for 15 days at a total volume of 1 mL/kg BW. The time that the first granulation tissue was observed, and the time that the wound was completely epithelialized and covered with granulation tissue were recorded. It was found that chlorogenic acid accelerated wound healing.

#### 2.3.2. Clinical Trials

Several human studies have been done to assess the antidiabetic activity of chlorogenic acid-rich foods and supplements, or pure chlorogenic acid. Johnston et al. [48] assessed the antidiabetic effects of dietary amounts of total CGAs in coffee. Nine participants were given 400 mL of water with 25 g of glucose and no coffee, caffeinated coffee (19.96, 10.65, and 10.60 mg/g of chlorogenic acid, 4-CQA, and 3-CQA, respectively), or decaffeinated coffee (11.91, 9.27, 8.26 mg/g of chlorogenic acid, 4-CQA, and 3-CQA, respectively). Both caffeinated and decaffeinated coffees decreased significantly (*p* < 0.05) the glucose-dependent insulinotropic polypeptide (GIP), suggesting a decrease in the rate of intestinal absorption of glucose. In addition, glucagon-like peptide 1 (GLP-1) secretion significantly increased 0–120 min postprandially after decaffeinated coffee consumption compared to the control. The results suggested that chlorogenic acid might have an antagonistic effect on glucose transport.

Thom [45] investigated the antidiabetic effects of chlorogenic acid in 12 subjects (six males and six females), which were given 400 mL of water with 25 g of sucrose and 0 g of coffee, 10 g of caffeinated instant coffee, 10 g of decaffeinated instant coffee, or 10 g of Coffee Slender^®^. Glucose absorption was evaluated during a 2-h oral glucose tolerance test (OGTT). The area under the curve (AUC) for plasma glucose concentration was significantly reduced by 6.9% (*p* < 0.05) with Coffee Slender^®^, while no significant changes were observed with normal caffeinated or decaffeinated coffees.

Iwai et al. [49] assessed the antihyperglycemic effects of an extract of decaffeinated green coffee beans, which was rich in CGAs. The study was done on 45 subjects (both sexes) who were 20–50 years old. The subjects were divided in three groups, where each group ingested 200 g of *Onigiri* (carbohydrate-rich rice snack) together with the test beverage (100 or 300 mg extract/200 mL water, or 200 mL water alone). At the end, 41 subjects (22 men and 19 women) were further analyzed. It was observed that plasma glucose was significantly lower (*p* < 0.05) 30 min after ingestion of the beverage with 300 mg extract. No significant differences were observed in plasma insulin profiles or plasma glucose AUC from 0 to 120 min. Then the group with highest mean postprandial glucose level (10 men and 8 women) 30 min after consumption of the placebo food was analyzed. It was found that plasma glucose was significantly lower (*p* < 0.01) after ingestion of both 100 and 300 mg extract beverages (~7% and 8% decrease, respectively). Moreover, the plasma glucose AUC from 0 to 120 min was significantly reduced (*p* < 0.05) following ingestion of the 100-mg extract beverage, but no significant changes in plasma insulin profiles were observed.

Ahrens and Thompson [50] studied the antidiabetic properties of Emulin™, a patented blend of chlorogenic acid, myricetin, and quercetin. The trial was performed on 40 subjects with type 2 diabetes divided in four groups: placebo/no medication, Emulin™/no medication, placebo/metformin, Emulin™/metformin. A capsule of 250 mg containing either Emulin™ or placebo was given 15 min before an OGTT, and 15 min before breakfast, lunch, and dinner every day for one week. The Emulin™/metformin group showed the most significant reduction in fasting blood, 2 h postprandial, AUC (*p* < 0.05) and actual peak glucose (*p* < 0.10) up to ~20%. The authors concluded that if Emulin^TM^ is consumed regularly could not only have the acute effect of lowering the glycemic impact of foods, but also chronically lower the blood glucose levels of type 2 diabetics.

Finally, van Dijk et al. [47] gave to 15 overweight men 12 g of decaffeinated coffee (1 g of chlorogenic acid, 500 mg of trigonelline, or 1 g of mannitol dissolved in 270 mL of water) to analyze the effects on glucose and insulin concentrations during a 2-h OGTT. Chlorogenic acid significantly reduced (*p* < 0.05) glucose and insulin concentrations (−0.7 mmol/L and −73 pmol/L, respectively) 15 min following the OGTT. However, none of the treatments affected insulin or glucose AUC.

### 2.4. Hypertension

#### 2.4.1. In Vivo Studies

Suzuki et al. [43] evaluated the effect of chlorogenic acid in spontaneously hypertensive rats (SHR). In a first experiment, rats were divided into groups of 4 or 5 and a single oral administration of chlorogenic acid was given to them by gavage. SHR were given 30, 100, 300, or 600 mg/kg BW of chlorogenic acid dissolved in physiologic saline. In a second experiment, rats were organized in groups of 6 and fed with a diet containing 0.5% chlorogenic acid for eight weeks. Wistar-Kyoto rats (WKY) were used as a control. Single ingestion of chlorogenic acid significantly reduced (*p* < 0.05) blood pressure in SHR with a minimal dose of 100 mg/kg BW. Furthermore, the development of hypertension was inhibited when SHR were fed the diet with 0.5% of chlorogenic acid for eight weeks. In contrast, chlorogenic acid had no effect on WKY rats.

#### 2.4.2. Clinical Trials

Studies done in humans have revealed that pure chlorogenic acid and chlorogenic-acid rich foods can have positive effects on blood pressure. Kozuma et al. [53] analyzed the anti-hypertension properties of green coffee extract (GCE). The study was made to 117 healthy males with mild hypertension. For 28 days subjects were given an instant soy sauce-flavored soup with 0, 46, 93, or 185 mg of green coffee extract (GCE) per day, which was equivalent to 0, 25, 50 and 100 mg of chlorogenic acid per day, respectively. Significant reductions of systolic blood pressure (SBP) and diastolic blood pressure (DBP) were observed (*p* < 0.05; −4.7 and −3.2 mmHg respectively) at a minimal dose of 93 mg GCE/day (50 mg chlorogenic acid/day). The authors suggested that the results could have been due to the antioxidant properties of chlorogenic acid (the main component of GCE) by improving endothelial dysfunction and reducing blood pressure. Watanabe et al. [54] also studied the anti-hypertension properties of GCE. The analysis was done on 28 subjects (11 males and 17 females), which were divided in two groups. The first group was given 125 mL/day of a fruit and vegetable juice containing 0.48 g of GCE (equivalent to 140 mg CGAs/day), while the second was given the juice without GCE. The study was done for 12 weeks. SBP and DBP were significantly reduced by 6.9% and 7.7%, respectively (*p* < 0.05) in patients that consumed juice with GCE.

Mubarak et al. [51] investigated the acute effects of chlorogenic acid on nitric oxide status, endothelial function, and blood pressure. A total of 23 healthy subjects (four men and 19 women) were given water (control) and 400 mg of chlorogenic acid dissolved in 200 mL of low nitrate water. Relative to the control, systolic and diastolic blood pressures were significantly lower (−2.41 and −1.53 mmHg, respectively; *p* < 0.05) with the chlorogenic acid treatment. Nitric oxide status and endothelial function were not significantly influenced. On the other hand, Ward et al. [52] analyzed the acute effect of two doses of chlorogenic acid on endothelial function and blood pressure. In the study, 16 healthy subjects (6 men and 10 women) received 0, 450, or 900 mg of chlorogenic acid, or 200 mg of (−)-epicatechin in random order 1 week apart. Only 14 participants completed the four treatments. No significant effect was found on blood pressure or peak flow-mediated dilation with any of the treatments. Nevertheless, there was a significant improvement in continuous flow-mediated dilation. Both 450 and 900 mg of chlorogenic acid resulted in significantly higher (*p* < 0.05) continuous flow-mediated dilation (0.47% and 0.65%, respectively) at 1 h, and significantly higher with 900 mg of chlorogenic acid (0.44%) at 4 h.

### 2.5. Metabolic Syndrome

#### 2.5.1. In Vivo Studies

Few studies have been done recently to assess the effect of chlorogenic acid in metabolic syndrome as a whole with promising results. Ma et al. [44] evaluated the preventive and therapeutic effects of chlorogenic acid on obesity and obesity-related liver steatosis and insulin resistance in mice. The mice were organized in groups of five. Two sets of experiments were done. In set 1, C57BL/6 mice were fed a normal diet or a high-fat diet for 15 weeks with two intraperitoneal injections of chlorogenic acid (100 mg/kg BW) or dimethyl sulfoxide (DMSO) per week. In set 2, obese mice were treated intraperitoneally with chlorogenic acid (100 mg/kg BW) or DMSO twice weekly for 6 weeks. Chlorogenic acid was effective in preventing weight gain, inhibiting development of liver steatosis, and blocking insulin resistance induced by high-fat diet. Furthermore, chlorogenic acid treatment in obese mice did not yield weight loss, but improved insulin sensitivity and reduced lipid accumulation in the liver.

#### 2.5.2. Clinical Trials

Patti et al. [35] evaluated the impact of Kepar (a natural supplement containing chlorogenic acid, *Curcuma longa*, silymarin, guggul, and inulin) on metabolic syndrome in humans. A total of 78 patients (45 men and 33 women) with metabolic syndrome were given two pills of Kepar per day for 4 months as add-on therapy to the ongoing treatment (antihypertensive, lipid-lowering, and hypoglycemic therapies). Significant reductions (*p* < 0.05) in body weight, body mass index, waist circumference, fasting glucose, and total cholesterol were found (~2%, 1%, 3%, 1.5%, 6% decrease, respectively) with Kepar supplementation.

**Figure 2:**
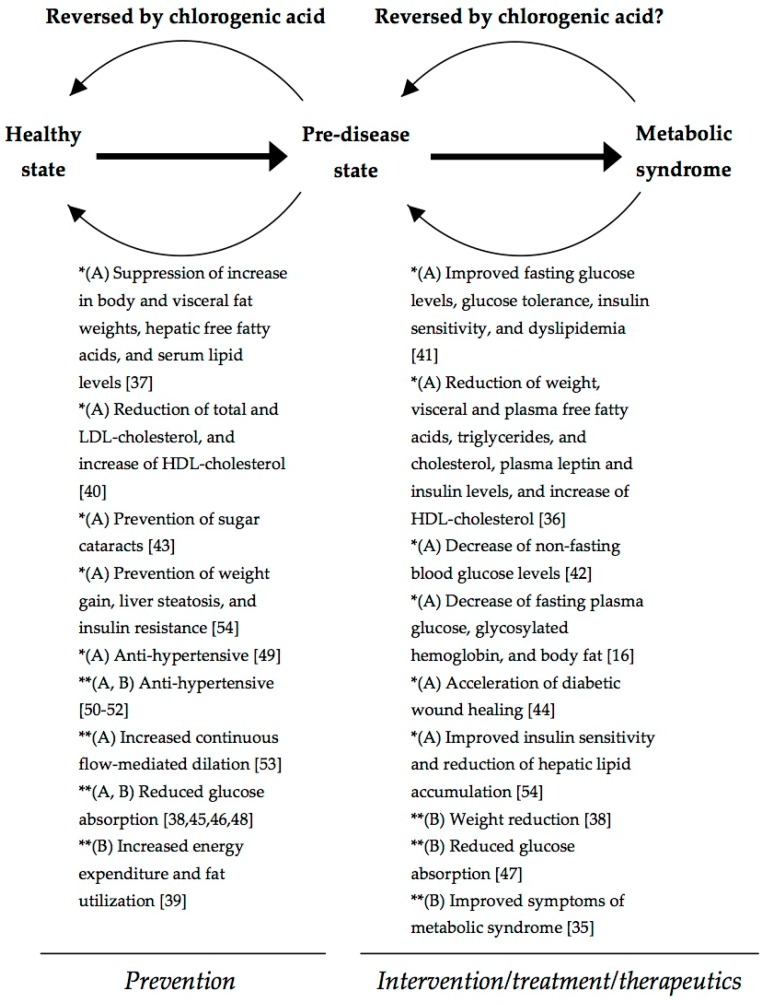
Proposed model for preventive and therapeutic effects of chlorogenic acid (A) or chlorogenic acid-rich foods and supplements (B) over metabolic syndrome based on in vivo studies (*) and clinical studies (**).

Recently we proposed a model of preventive and therapeutic effects of food juices on chronic diseases based on in vivo and clinical studies [55,56]. Similarly, in the present review Figure 2 summarizes the preventive and therapeutic effects of chlorogenic acid and chlorogenic-acid rich foods and supplements over metabolic syndrome based on the in vivo and clinical studies discussed above. According to these studies, chlorogenic acid can prevent a pre-disease state of metabolic syndrome, allowing return to a healthy state. Likewise, literature indicates that chlorogenic acid could be used as a therapeutic agent against metabolic syndrome. However, as it can be appreciated in Figure 2, most clinical studies have been done with chlorogenic acid-rich foods (mainly coffee) and supplements; thus, the observed positive effects on human health could be caused by chlorogenic acid itself or by the combination of bioactive compounds present in these foods and supplements. On the other hand, the clinical studies done with pure chlorogenic acid are very limited, which report some positive effects, particularly in hypertension. Therefore, more clinical studies using chlorogenic acid in its pure form are needed to assess its full potential as a preventive/therapeutic agent against metabolic syndrome.

### 2.6. Mechanisms of Action

**Figure 3:**
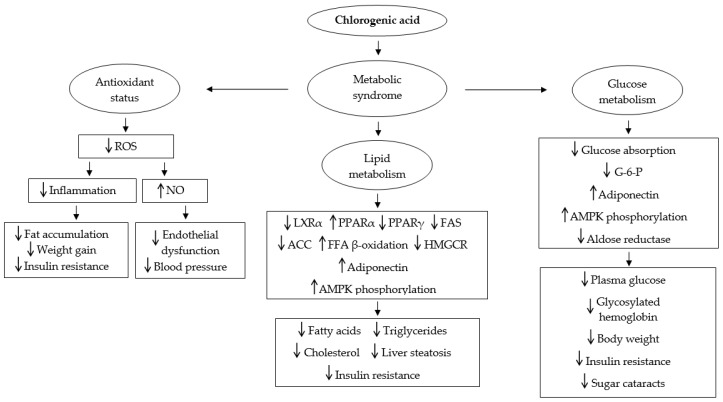
Mechanisms of action of chlorogenic acid over metabolic syndrome. Abbreviations: ACC = acetyl-CoA carboxylase, AMPK = AMP-activated protein kinase, FAS = fatty acid synthase, FFA = free fatty acid, G-6-P = glucose-6-phosphatase, HMGCR = 3-hydroxy-3-methylglutaryl CoA reductase, LXRα = liver X receptor α, NO = nitric oxide, PPARα = peroxisome proliferator-activated receptor α, PPARγ = peroxisome proliferator-activated receptor γ, ROS = reactive oxygen species.

Several mechanisms have been proposed to explain how chlorogenic acid exerts its positive effects over metabolic syndrome. The mechanisms are summarized in Figure 3. Some of these effects have been associated to the antioxidant and anti-inflammatory properties of chlorogenic acid. Oxidative stress in accumulated fat has been suggested as an early instigator of obesity-associated metabolic syndrome [34]. Also, chronic inflammation has been associated with metabolic syndrome [57]. Ma et al. [44] found that chlorogenic acid treatment in mice fed with a high-fat diet and obese mice greatly reduced expression of macrophage marker genes in adipose tissue including F4/80, Cd68, Cd11b and Cd11c, and pro-inflammatory mediator genes including TNF-α and MCP-1 in macrophages. In addition, the authors observed that chlorogenic acid inhibited hepatic peroxisome proliferator-activated receptor γ (PPARγ), which promotes fatty acid uptake into liver cells. Therefore, the mechanism proposed was that chlorogenic acid scavenges reactive oxygen species (ROS) generated by consumption of high-fat diet, which suppresses the expression of inflammation, and consequently reduces fat accumulation, weight gain, and insulin resistance, while inhibition of PPARγ prevents and improves liver steatosis.

Furthermore, chlorogenic acid has been observed to have impact over important transcription factors and enzymes that regulate lipid metabolism, which has been associated to positive effects on obesity and dyslipidemia. Hepatic lipid metabolism plays a central role in whole-body lipid metabolism through the opposing activities of liver X receptor α (LXRα) and peroxisome proliferator-activated receptor α (PPARα), which are nuclear receptor transcription factors that are highly expressed in the liver. On one hand, LXRα regulates fatty acid and triglyceride synthesis through the direct activation of genes encoding lipogenic enzymes, including fatty acid synthase (FAS) and acetyl-CoA carboxylase (ACC); on the other hand, PPARα enhances fatty acid degradation through β-oxidation, which results in a reduction of hepatic triglyceride content, and consequently in an increase in insulin sensitivity [37]. Cho et al. [36] found that chlorogenic acid supplementation in high-fat diet-induced-obese mice significantly inhibited fatty acid synthase (FAS), and increased fatty acid β-oxidation activity and peroxisome proliferator-activated receptor α (PPARα) expression compared to the high-fat control group. Moreover, Huang et al. [37] showed that chlorogenic acid down-regulated LXRα mRNA expression with corresponding reductions in ACC and FAS mRNA levels, and up-regulated PPARα mRNA expression in the liver in a dose-dependent manner. Therefore, it was concluded that the observed reductions in serum and hepatic lipids in rats under a high-fat diet burden was caused by increase in PPAR𝛼 expression leading to accelerated fatty acid β-oxidation, and by decrease in LXRα expression which resulted in a reduction in lipid synthesis. Increase in PPARα expression by chlorogenic acid has been also implicated in improvement of dyslipidemia in late diabetic mice [16]. As for hypercholesterolemia, chlorogenic acid has been found to inhibit 3-hydroxy-3-methylglutaryl CoA reductase (HMGCR) [36], a key enzyme in cholesterol biosynthesis, which helps explain the observed reductions in cholesterol.

The anti-obesity and antidiabetic properties of chlorogenic acid have been attributed to its impact in glucose metabolism as well. Thom [45] proposed that decrease in glucose absorption and weight reduction effects observed in humans that consumed chlorogenic acid enriched instant coffee could be caused by chlorogenic acid through inhibition of glucose absorption in the small intestine by inhibiting glucose-6-phosphate translocase 1 [44] and glucose release by inhibiting hepatic glucose-6-phosphatase activity. This would cause a decrease of glucose in the general circulation, and consequently less insulin activity. Reduced availability of glucose as an energy source would lead then to consumption of fat reserves, while less insulin activity would lead to a reduction in fewer fatty deposits in adipose tissue. The proposed mechanism is supported by the fact that chlorogenic acid can be found intact in the small intestine of humans [58]. In addition, it has been found that chlorogenic acid decreases the expression of hepatic glucose-6-phosphatase, and increases adiponectin receptors, adiponectin, and phosphorylation of AMP-activated protein kinase (AMPK) in late diabetic mice [16]. Reduction of glucose-6-phosphatase activity, and increase in adiponectin receptors, adiponectin, and AMPK phosphorylation have been associated with decrease of fasting glucose, glycosylated hemoglobin, triglycerides, cholesterol, and hepatic steatosis, and increase of insulin sensitivity and glucose tolerance [16,39]. In the case of sugar cataracts, it has been found that chlorogenic acid prevents them through inhibition of aldose reductase activity, an enzyme that in diabetic conditions transforms excess glucose into sorbitol, which accumulates in the cells due to its low permeability, causing lens fiber degeneration and opacification [41]. On the other hand, the hypotensive effect of GCE observed in humans has been attributed to chlorogenic acid, as it is the main component of the extract studied [53]. In hypertensive patients, it has been observed increased oxidative stress, such as elevated levels of hydrogen peroxide and superoxide anions, and reduced antioxidant mechanisms in hypertensive patients [59,60]. Superoxide anions decrease nitric oxide bioavailability in endothelial tissue, and nitric oxide deficiency has been proposed as a cause of hypertension [61]. Furthermore, it has been found that antioxidant vitamins, such as ascorbic acid, reduce blood pressure [62] and improve endothelium-dependent vasodilation in hypertensive patients by neutralizing ROS and restoring nitric oxide activity [63]. Therefore, Kozuma et al. [53] proposed that the antioxidant properties of chlorogenic acid could be partly responsible for improving endothelial dysfunction and reducing blood pressure.

Caution should be taken when interpreting the effects observed by ingestion of chlorogenic acid. Only about one-third of the ingested chlorogenic acid is absorbed from the small intestine into the bloodstream in humans, while the rest reaches the colon and it is extensively metabolized by the colonic microbiota, resulting in compounds such as hippuric acid, caffeic acid, and ferulic acid [58,64]. Therefore, it is possible that the positive effects of chlorogenic acid on human health could be due to these metabolites. For instance, it has been suggested that ferulic acid, one of the metabolites of chlorogenic acid, could be the cause of the hypotensive effects observed in humans after consumption of GCE [54], as it is known that ferulic acid can scavenge superoxide anions [65] and has shown hypotensive effects in rats [66]. Hence, more studies will be needed to determine whether the health benefits found after ingestion of chlorogenic acid are caused directly by this compound or its metabolites. For more information on the pharmacokinetics of chlorogenic acid, see the recent review of Liang and Kitts [20].

## 3. The Role of Chlorogenic Acid as a Food Additive

### 3.1. Antimicrobial Properties

The use of synthetic chemical preservatives in food products is a concern for consumers, and therefore the identification of consumer acceptable natural antimicrobials has become valuable [67]. Several studies have confirmed that chlorogenic acid could be used as an antimicrobial agent (Table 3), which could be useful for the preservation of food products.

Chlorogenic acid has activity against a wide range of microorganisms, including bacteria, yeasts, molds, viruses, and amoebas. Most studies have been done on bacteria, and the majority of microorganisms studied have been pathogens. Among the Gram-negative bacteria, chlorogenic acid has shown activity against *Escherichia coli* (*E. coli*), *E. coli* O157:H7, *Klebsiella pneumoniae*, *Proteus vulgaris*, *Pseudomonas aeruginosa*, *Salmonella typhimurium*, *Shigella dysenteriae*, and *Stenotrophomonas maltophilia* with minimum inhibitory concentrations (MICs) ranging from 0.008 to 10 mg/mL [8,12,67,68]. Furthermore, chlorogenic acid has inhibited growth of the Gram-positive bacteria *Bacillus cereus*, *Bacillus subtilis*, *Enterococcus faecalis*, *Enterococcus faecium*, *Listeria innocua*, *Staphylococcus aureus*, Methicillin-resistant *Staphylococcus aureus* (MRSA), and *Streptococcus pneumoniae* with MICs of 0.02–10 mg/mL [9,12,19,67,68,70]. Moreover, inhibited yeasts by chlorogenic acid include *Candida albicans*, *Malassezia furfur*, *Saccharomycses cerevisiae*, and *Trichosporon beigelii* with MICs ranging from 0.04 to 10 mg/mL [67,68,71]. In addition, chlorogenic acid has caused 73% inhibition to the mold *Penicillium chrysogenum* at a concentration of 25 mM (8.86 mg/mL) [67]. Also, chlorogenic acid has shown 50% inhibition (IC_50_) to the hepatitis B virus extracellular DNA replication, intracellular DNA replication, and surface antigen at concentrations of 1.2, 1.3 and 241.5 μM (0.4, 0.5 and 85 μg/mL), respectively [73], as well as an IC_50_ of 6.3 μg/mL in Enterovirus 71 [72]. Finally, chlorogenic acid has shown activity against encystment of the *amoeba Acanthamoeba triangularis* at a concentration of 1 mg/mL [74].

Antimicrobial activity of chlorogenic acid has been tested as well in probiotic bacteria, which are beneficial to human health. Puupponen-Pimiä et al. [69] found that chlorogenic acid did not inhibit *Bifidobacterium lactis*, *Lactobacillus crispatus* (*L. crispatus*), *L. johnsonii*, *L. paracasei*, *L. plantarum*, *L. reuteri*, nor *L. rhamnosus* (including the GG strain) at concentrations up to 10 mg/mL. As suggested by Muthuswamy and Vasantha-Rupasinghe [67], the resistance of these probiotics is encouraging towards the potential use of chlorogenic acid as a natural antimicrobial compound in value-added food products.

The differences in the sensitivity of microorganisms against chlorogenic acid could be attributed in part to differences in their cell wall structure that lead to differences in permeability. For instance, some bacteria like *E. coli* have porins that allow the passage of certain compounds, while other bacteria such as *Enterobacter aerogenes* lack porins [67]. Therefore, it has been proposed that microorganisms that are permeable to chlorogenic acid are more sensitive than those that are not permeable [67].

The mechanism by which chlorogenic acid exerts its antimicrobial activity is not yet fully understood. Some studies, however, indicate that chlorogenic acid acts by mainly disrupting the cell membrane of the microorganism, leading to its death. Sung and Lee [71] elucidated the antifungal mode of action of chlorogenic acid by using flow cytometry and analyzing membrane dynamics in *Candida albicans*. Results suggested that chlorogenic acid may exert antifungal activity by disrupting the cell membrane, possibly by perturbing the lipid bilayers, causing leakage of ions and other materials, formation of pores, and dissipation of the membrane potential. In addition, Lou et al. [12] studied the antibacterial mechanism of chlorogenic acid using *Shigella dysenteriae* and *Streptococcus pneumoniae* as models. The authors concluded that chlorogenic acid killed the bacteria by provoking irreversible permeability changes in the cell membrane, causing disruption of the membrane potential and loss of cytoplasm macromolecules including nucleotides. Finally, Li et al. [9] investigated the antimicrobial mechanism of chlorogenic acid against *Staphylococcus aureus* by examining the cell membrane structure under electron microscope, and by measuring intracellular and extracellular ATP concentrations, membrane potential, intracellular pH, and release of cell components. The researchers observed a decrease in the intracellular ATP concentrations, although no proportional increase in extracellular ATP; increase in the release of cell components, intracellular pH reduction, cell membrane hyperpolarization, and damage of the cell membrane, leading to the conclusion that chlorogenic acid destroys the permeability of the cell membrane.

### 3.2. Antioxidant Activity

Chlorogenic acid has been found to prevent certain undesirable chemical changes that can take place in food, such as lipid oxidation. Lipid-based foodstuffs are prone to oxidation induced and mediated by free radicals, photon impact, and/or transition metals [75]. The oxidation of lipids leads to the development of undesirable off-flavors and potentially toxic compounds (e.g., malondialdehyde and 4-hydroxynonenal), and can also influence food quality parameters like texture, appearance, taste (rancidification), and nutritional profile (loss of vitamins and essential fatty acids) [76,77]. Studies have shown that the antioxidant capacity of chlorogenic acid can counteract oxidation of emulsified lipids. Laguerre et al. [76] investigated the antioxidant capacity of chlorogenic acid and several of its alkyl esters (C1, C4, C8, C12, C16, C18 and C20) in emulsified tung oil using the conjugated autoxidizable triene assay, which uses 2,2′-azobis(2-amidinopropane) dihydrochloride (AAPH) to induce oxidation. It was observed that chlorogenic acid and its alkyl esters inhibited AAPH-mediated oxidation of tung oil with improved antioxidant capacity until an alkyl chain length of 12 carbons and collapse of antioxidant capacity with larger alkyl chains. It was suggested that the observed increase in antioxidant capacity until C_12_ could be due to closer location to the oil droplet, while collapse of antioxidant capacity with larger alkyl chains could be due to formation of aggregates, possibly micelles. In addition, Sasaki et al. [77] studied the ability of chlorogenic acid and some of its alkyl esters (C_4_, C_8_ and C_12_) to inhibit lipid oxidation in oil-in-water emulsions. Menhaden oil was used as model lipid. Chlorogenic acid and its alkyl esters were added to the emulsions at concentrations ranging from 47 to 120 µM. Emulsions were placed into headspace vials, sealed with polytetrafluoroethylene (PTFE)/butyl rubber septa, and allowed to oxidize in the absence of light at 20 °C for up to 7 days. Headspace propanol and lipid hydroperoxide were measured. Chlorogenic acid and its C_4_ and C_8_ esters inhibited propanol and lipid hydroperoxide formation with greater antioxidant activity with increasing chain length, while C12 was ineffective. It was suggested that chlorogenic acid and its esters inhibit lipid oxidation by a combination of free radical scavenging in the lipid phase and metal chelation in the aqueous phase.

### 3.3. Conservation of Other Bioactive Compounds

In addition, chlorogenic acid can inhibit the degradation of other natural bioactive phytochemicals found in foodstuffs, such as anthocyanins. Kopjar et al. [78] investigated the prevention of degradation of anthocyanins in blackberry juice by addition of sugars and chlorogenic acid. Samples were kept in glass bottles and stored for 90 days in the dark at 4 °C. The authors observed that chlorogenic acid alone decreased anthocyanin degradation through a copigmentation mechanism. The effect lasted for the entire storage period, and was enhanced by combining chlorogenic acid with glucose or trehalose. More studies should be done to determine if chlorogenic acid can inhibit the degradation of other bioactive compounds besides anthocyanins.

### 3.4. Prebiotic Properties

Another growing area of food research are prebiotics, which are food components that stimulate the growth of beneficial bacteria in the human intestine microbiota. Chlorogenic acid has shown activity as a prebiotic. Mills et al. [79] used a stirred, anaerobic, pH-controlled, batch culture fermentation model to mimic the distal region of the human colon in order to investigate the effect of coffee and chlorogenic acid on the growth of human fecal microbiota. The coffee with the highest level of CGAs (high-CGAs coffee) induced a significant increase (*p* < 0.05) in the growth of Bifidobacterium spp. Pure chlorogenic acid in a quantity that matched the level of CGAs found in one cup of high-CGAs coffee (80.8 mg) induced a significant increase in the growth of Bifidobacterium spp. as well as *Clostridium coccoides-Eubacterium rectale* group, the latter group having also potential to benefit human health. However, this study was an in vitro test. Studies in humans are needed to evaluate the full potential of chlorogenic acid as a prebiotic.

## 4. Conclusions and Future Research Topics

Chlorogenic acid has multifunctional properties as a nutraceutical and food additive. As a nutraceutical, chlorogenic acid has anti-oxidant, anti-inflammatory, anti-obesity, antidyslipidemia, antidiabetic, and antihypertensive properties, which can serve for the prevention and treatment of metabolic syndrome and associated disorders. On the other hand, as a food additive, chlorogenic acid has antimicrobial activity against a wide range of microorganisms, inhibits lipid oxidation, prevents degradation of other bioactive compounds, and can function as a prebiotic. The double role of chlorogenic acid as a nutraceutical and food additive makes it an excellent candidate for the formulation of dietary supplements and functional foods. Most clinical studies have been done with coffee [80]; therefore, more clinical studies with pure chlorogenic acid are needed to assess its full potential against metabolic syndrome. Also, more studies must be done to determine whether chlorogenic acid or its metabolites are indeed responsible for the observed positive effects in human health. Moreover, the application of chlorogenic acid as a food additive is a new area of research; hence, additional studies are needed to determine necessary concentrations to use chlorogenic acid as a preservative and prebiotic in foods, the cost-effectiveness of using it instead of traditional preservatives, its potential to prevent the degradation of other bioactive compounds, and its prebiotic activity in humans. Regarding the cost-effectiveness of using chlorogenic acid instead of traditional preservatives, some advances have been made. New processes are being designed for the extraction and purification of chlorogenic acid with economically attractive costs estimated in the range of 0.06 – 0.38 USD/mg [81]. Therefore, it is likely that chlorogenic acid will have competitive costs in the near future, which will allow it to replace traditional preservatives.

## Figures and Tables

**Table 1:** In vivo studies evaluating the effect of chlorogenic acid on the prevention and treatment of metabolic syndrome and associated disorders.

Disorder	Animal Model (N = Total Number of Animals)	Study Details	Experimental Findings	Refs.
Obesity	Male induced-obese ICR mice (N = 32)	Mice were fed a normal diet, high-fat diet, high-fat diet with chlorogenic acid (0.2 g/kg diet), or high-fat diet with caffeic acid (0.2 g/kg diet) for 8 weeks.	Both chlorogenic acid and caffeic acid significantly reduced body weight, visceral fat mass, plasma leptin and insulin levels, triglycerides in liver and heart, and cholesterol in adipose tissue and heart compared to the high-fat control group. Only chlorogenic acid significantly lowered triglyceride contents in adipose tissue.	[36]
	Male Sprague-Dawley rats (N = 40)	Rats were fed a normal diet, a high-fat diet, or a high-fat diet with chlorogenic acid (20 or 90 mg/kg BW) for 12 weeks.	Chlorogenic acid suppressed increases in body and visceral fat weights, and hepatic free fatty acids.	[37]
Dyslipidemia	Male induced-obese ICR mice (N = 32)	Mice were fed a normal diet, high-fat diet, high-fat diet with chlorogenic acid (0.2 g/kg diet), or high-fat diet with caffeic acid (0.2 g/kg diet) for 8 weeks.	Both chlorogenic acid and caffeic acid significantly reduced plasma free fatty acids, triglycerides, and cholesterol, and significantly increased HDL-cholesterol/total cholesterol ratio compared to the high-fat control group.	[36]
	Male Sprague-Dawley rats (N = 40)	Rats were fed a normal diet, a high-fat diet, or a high-fat diet with chlorogenic acid (20 or 90 mg/kg BW) for 12 weeks.	Chlorogenic acid suppressed increases in serum lipid levels.	[37]
	Male Sprague-Dawley rats (N = 40)	Rats were fed a normal diet, a high-cholesterol diet, or a high-cholesterol diet with chlorogenic acid (1 or 10 mg/kg BW/day). Chlorogenic acid was administered orally by gavage (20 mL/kg BW) once per day for 28 days.	Chlorogenic acid (10 mg/kg) significantly reduced total and LDL-cholesterol, increased HDL cholesterol, and improved both the atherogenic index and the cardiac risk factor.	[38]
Diabetes	*Lepr^db/db^* mice (N = 24)	Mice were treated with vehicle, 250 mg/kg BW oral gavage metformin or 250 mg/kg BW chlorogenic acid intraperitoneally daily for 2 weeks.	Chlorogenic acid inhibited hepatic glucose-6-phosphatase expression and activity, decreased hepatic steatosis, improved lipid profiles and skeletal muscle glucose uptake, which improved fasting glucose levels, glucose tolerance, insulin sensitivity and dyslipidemia in the *Lepr^db/db^* mice.	[39]
	Streptozotocin-induced Sprague-Dawley diabetic rats (N = 6 or 7 per group)	Rats were orally treated once a day for 11 days with mulberry extract (250 or 750 mg/kg BW), chlorogenic acid (9 or 27 mg/kg BW), rutin (5 or 15 mg/kg BW), or isoquercetin (3 or 9 mg/kg BW) suspended in 0.25% of methylcellulose using a dosing volume of 5 mL/kg BW for all treatments.	Mulberry extract, chlorogenic acid (27 mg/kg) and rutin decreased non-fasting blood glucose levels.	[40]
Diabetes	Female C57BL/BKS late diabetic mice (N = 32).	Mice were divided in four groups: *db/m*-control group, *db/m*-chlorogenic acid group, *db/db*-control group, and *db/db-*chlorogenic acid group. Animals in chlorogenic acid groups were given chlorogenic acid (80 mg/kg BW) for 12 weeks.	Chlorogenic acid significantly decreased percentage of body fat, fasting plasma glucose, and HbA1c compared to the *db/db*-control group.	[16]
	Galactose-induced sugar cataract Sprague-Dawley rat model (N = 40).	Rats were fed a normal diet, 50% galactose diet, or 50% galactose diet with chlorogenic acid (10 or 50 mg/kg BW) for 2 weeks.	Chlorogenic acid prevented the development of sugar cataracts.	[41]
	Streptozotocin-induced Sprague-Dawley diabetic male rats (N = 40).	Rats were administered intraperitoneally 50 mg/kg BW of chlorogenic or PBS for 15 days at a total volume of 1 mL/kg BW.	Chlorogenic acid accelerated wound healing, i.e., it shortened the time needed for the wound to be completely epithelialized and covered with granulation tissue.	[42]
Hypertension	Spontaneously hypertensive rats (N = 45)	Experiment 1: chlorogenic acid was given to rats in a single dose orally by gavage (30, 100, 300, or 600 mg/kg BW). Experiment 2: rats were fed with a diet containing 0.5% chlorogenic acid for 8 weeks.	Single ingestion of chlorogenic acid significantly reduced blood pressure with a minimal dose of 100 mg/kg. Development of hypertension was inhibited when rats were fed the diet with 0.5% chlorogenic acid for 8 weeks.	[43]
Metabolic syndrome	Male C57BL/6 mice (N = 5 per group)	Experiment 1: mice were fed a normal diet or a high-fat diet for 15 weeks with two intraperitoneal injections of chlorogenic acid (100 mg/kg BW) or DMSO per week. Experiment 2: obese mice were treated intraperitoneally with chlorogenic acid (100 mg/kg BW) or DMSO twice weekly for 6 weeks.	Chlorogenic acid prevented weight gain, inhibited development of liver steatosis, and blocked insulin resistance induced by high-fat diet. Treatment with chlorogenic acid in obese mice did not yield weight loss, but improved insulin sensitivity and reduced lipid accumulation in the liver.	[44]

Abbreviations: BW = body weight, DMSO = dimethyl sulfoxide, HbA1c = glycosylated hemoglobin, ICR = imprinting control region, HDL = high-density lipoprotein, LDL = low-density lipoprotein, PBS = phosphate-buffered saline.

**Table 2:** Clinical trials evaluating the effect of chlorogenic acid or chlorogenic acid-rich foods and supplements on the prevention and treatment of metabolic syndrome and associated disorders.

Disorder	Chlorogenic Acid Source	Subjects (N = Total Number of Subjects)	Study Details	Experimental Findings	Refs.
Obesity	Coffee Slender^®^	Overweight subjects, BMI 27.5–32 kg/m^2^ (N = 30)	Half volunteers drank Coffee Slender^®^ (45 mg CGAs/g), other half drank normal instant coffee (30–40 mg CGAs/g), 5 cups/day (11 g of coffee/day) for 12 weeks.	Weight was significantly reduced (−5.4 kg) with Coffee Slender^®^.	[45]
	CGAs-rich beverage	Healthy men, 36.1 ± 7.4 years old (N = 18)	Subjects consumed 185 mL of a test beverage with or without CGAs (329 mg) daily for 4 weeks.	CGAs beverage significantly increased postprandial energy expenditure and fat utilization.	[46]
Diabetes	Pure chlorogenic acid	Overweight men, BMI 25–35 kg/m^2^ (N = 15)	Subjects were given 12 g decaffeinated coffee, 1 g chlorogenic acid, 500 mg trigonelline, or 1 g mannitol dissolved in 270 mL water.	Chlorogenic acid significantly reduced glucose and insulin concentrations (−0.7 mmol/L and −73 pmol/L, respectively) 15 min following an OGTT.	[47]
	Instant coffee	Healthy subjects, 26 ± 3.2 years old, 4 men and 5 women, (N = 9)	Subjects consumed one of three 400-mL beverages: caffeinated coffee (40 mg CGAs/g), decaffeinated coffee (30 mg CGAs/g), or glucose dissolved in water.	Both caffeinated and decaffeinated coffee beverages decreased significantly the GIP. Decaffeinated coffee increased significantly GLP-1.	[48]
	Coffee Slender^®^	Healthy subjects, BMI < 25 kg/m^2^, 6 men and 6 women (N = 12)	Subjects were given 400 mL of water with 25 g sucrose and 0 g coffee, 10 g decaffeinated instant coffee (30 mg CGAs/g), 10 g caffeinated instant coffee (40 mg CGAs/g), or 10 g Coffee Slender^®^ (45 mg CGAs/g).	Glucose absorption was significantly reduced by 6.9% with Coffee Slender^®^.	[45]
	Decaffeinated GCE rich in CGAs	Healthy subjects with highest postprandial glucose levels, 10 men and 8 women (N = 18)	Subjects were given a carbohydrate-rich snack with 100 or 300 mg extract (13.9% chlorogenic acid DW) in 200 mL water, or 200 mL water alone.	Plasma glucose and AUC were significantly reduced with 100 mg extract beverage.	[49]
Diabetes	Emulin^TM^ (a patented blend of chlorogenic acid, myricetin, and quercetin)	Subjects with type 2 diabetes, 18+ years old, BMI ≥ 30 kg/m^2^ (N = 40)	Subjects divided in four groups of 10: placebo/no medication, Emulin^TM^/no medication, placebo/ metformin, Emulin™/ metformin. Capsule of 250 mg (placebo or Emulin™) was given 15 min before OGTT, and 15 min before breakfast, lunch, and dinner every day for one week.	The Emulin^TM^/metformin group showed the most significant reduction in fasting blood, 2 h postprandial, actual peak, and AUC glucose up to 20%.	[50]
Hypertension	Pure chlorogenic acid	Healthy subjects, 4 men and 19 women (N = 23)	Subjects were given water (control) and 400 mg of chlorogenic acid dissolved in 200 mL of low nitrate water.	SBP and DBP were significantly reduced (−2.41 and −1.53 mmHg, respectively) with chlorogenic acid treatment.	[51]
	Pure chlorogenic acid	Healthy subjects, both sexes (N = 14)	Subjects were given 0, 450, or 900 mg of chlorogenic acid, or 200 mg of (–)-epicatechin in random order 1 week apart.	No significant effect on BP or peak FMD. Significant increase in continuous FMD at 1 and 4 h with chlorogenic acid (900 mg).	[52]
	GCE	Men with mild hypertension, 30–50 years old (N = 117)	Subjects were given an instant soy sauce-flavored soup with GCE (equivalent to 0, 25, 50 and 100 mg chlorogenic acid) for 28 days.	Significant reductions of SBP and DBP (−4.7 and −3.2 mmHg, respectively) at minimal dose of 93 mg GCE/day.	[53]
	GCE	Subjects with mild hypertension, 11 men and 17 women (N = 28)	Half the subjects were given 125 mL/day of fruit and vegetable juice with 0.48 g GCE (140 mg CGAs/day), or the fruit and vegetable juice alone for 12 weeks.	SBP and DBP were significantly reduced by 6.9% and 7.7%, respectively with juice containing GCE.	[54]
Metabolic syndrome	Kepar (supplement with chlorogenic acid, *Curcuma longa*, silymarin, guggul, and inulin)	Subjects with metabolic syndrome, 45 men and 33 women (N = 78)	Subjects were given 2 pills Kepar/day for 4 months as add-on therapy.	Kepar treatment significantly reduced body weight, BMI, waist circumference, fasting glucose, and total cholesterol (~2%, 1%, 3%, 1.5%, 6% decrease, respectively).	[35]

Abbreviations: AUC = area under the curve, BMI = body mass index, BP = blood pressure, CGAs = chlorogenic acid and other related compounds (isomers, derivatives, and/or other quinic acid conjugates), DBP = diastolic blood pressure, DW = dry weight, FMD = flow-mediated dilation, GCE = green coffee extract, GIP = glucose-dependent insulinotropic polypeptide, GLP-1 = glucagon-like peptide 1, OGTT = oral glucose tolerance test, SBP = systolic blood pressure.

**Table 3:** Antimicrobial activity of chlorogenic acid against different microorganisms.

Microorganism Type	Species	Relevance	MIC (mg/mL)	References
Gram-negative bacteria	*Enterobacter aerogenes*	Pathogen	>8.86	[67]
	*Escherichia coli*	Pathogen	0.08 (MIC)–10 (MIC_80_)	[12,68]
	*Escherichia coli* O157:H7	Pathogen	3.54 (~90% inhibition)	[67]
	*Klebsiella pneumoniae*	Pathogen	5 (MIC_80_)	[68]
	*Proteus vulgaris*	Pathogen	10 (MIC_80_)	[68]
	*Pseudomonas aeruginosa*	Pathogen	10 (MIC_80_)	[68]
	*Salmonella typhimurium*	Pathogen	0.04	[12]
	*Shigella dysenteriae*	Pathogen	0.02	[12]
	*Stenotrophomonas maltophilia*	Pathogen	0.008–0.016	[8]
Gram-positive bacteria	*Bacillus cereus*	Pathogen	0.064	[19]
	*Bacillus subtilis*	Food spoiler	0.04	[12]
	*Bifidobacterium lactis*	Probiotic	>10	[69]
	*Enterococcus faecalis*	Pathogen	0.064	[19]
	*Enterococcus faecium*	Pathogen	10 (MIC_80_)	[68]
	*Lactobacillus crispatus*	Probiotic	>10	[69]
	*Lactobacillus johnsonii*	Probiotic	>10	[69]
	*Lactobacillus paracasei*	Probiotic	>10	[69]
	*Lactobacillus plantarum*	Probiotic	>10	[69]
	*Lactobacillus reuteri*	Probiotic	>10	[69]
	*Lactobacillus rhamnosus*	Probiotic	>10	[69]
	*Lactobacillus rhamnosus* GG	Probiotic	>10	[69]
	*Listeria innocua*	Surrogate for *Listeria monocytogenes*	3.54 (~90% inhibition)	[67]
	*Staphylococcus aureus*	Pathogen	0.04 (MIC)–10 (MIC_80_)	[9,12,19,68,70]
	Methicillin-resistant *Staphylococcus aureus* (MRSA)	Pathogen	0.5–5	[9,70]
	*Streptococcus pneumoniae*	Pathogen	0.02	[12]
Yeasts	*Candida albicans*	Pathogen	0.08 (MIC)–10 (MIC_80_)	[68,71]
	*Malassezia furfur*	Pathogen	0.04	[71]
	*Saccharomyces cerevisiae*	Food spoiler	8.86 (92% inhibition)	[67]
	*Trichosporon beigelii*	Pathogen	0.04	[71]
Molds	*Penicillium chrysogenum*	Food spoiler	8.86 (73% inhibition)	[67]
Viruses	Enterovirus 71	Pathogen	0.0063 (IC_50_)	[72]
	Hepatitis B virus	Pathogen	0.0004, 0.0005, 0.085 (IC_50_ extracellular and intracellular DNA replication, and surface antigen, respectively.)	[73]
Amoebas	*Acanthamoeba triangularis*	Pathogen	1 *	[74]

* Only one concentration was tested. Abbreviations: IC_50_ = Minimum concentration to inhibit 50% of cells, MIC = Minimum inhibitory concentration, MIC_80_ = Minimum concentration to inhibit 80% of cells, MRSA = Methicillin-resistant *Staphylococcus aureus*.
